# Beyond the snapshot: harnessing daily physical activity for heart failure characterization

**DOI:** 10.1007/s00392-024-02511-5

**Published:** 2024-08-21

**Authors:** Birgit Assmus, Pascal Bauer

**Affiliations:** 1 Department of Cardiology and Angiology, University of Giessen, Giessen, Germany; 2 Department of Cardiology and DZHK (German Centre for Cardiovascular Research), Partner Site Rhein/Main, Kerckhoff Heart Center, Bad Nauheim, Hessen Germany; 3 Med. Klinik I, Kardiologie / Angiologie, UKGM, Universitätsklinikum Gießen, Klinikstr. 33, 35392 Giessen, Germany

“Sirs:”

Heart failure (HF) has been characterized as a global pandemic, affecting an estimated 64.3 million individuals worldwide in 2017 [1]. Its prevalence is projected to rise due to improved survival rates following HF diagnosis, attributable to the availability of lifesaving, evidence-based treatments, and the overall increase in life expectancy. It is well established that even moderate amounts of physical activity (PA) are associated with a lower risk of HF-related hospital admissions, mortality and other health-related outcomes in patients with chronic HF [2, 3]. Therefore, an increasing number of intervention studies aim to increase physical activity in HF patients. Physical activity is defined as bodily movement produced by skeletal muscles that results in energy expenditure [4]. Physical activity has to be discriminated from functional capacity, which is only a snapshot of physical activity assessed, for example, with a standardized protocol during a 6-min walk test or cardiopulmonary exercise testing. Physical activity constitutes therefore a more holistic approach. Subjective measures of physical activity include self-reported questionnaires, but these are limited by subjectivity and recall, and, most importantly, overestimate physical activity up to 44% in men and 138% in women. Moreover, there is a substantial lack in precision to detect day-to-day changes in physical activity. Objective methods to assess physical activity comprise direct assessments of one or several dimensions of physical activity such as frequency, intensity, duration and type of activity, like direct step count. Due to the development of activity monitors and wearables, the direct and objective assessment of physical activity has become widely available not only for research purposes in health and disease, but also for lifestyle modification and during rehabilitation.

However, whether regular physical activity tracking can be applied for training guidance in health and disease is so far unsure. First, there is an urgent need for characterizing different disease stages and their association with physical activity.

Within this context, the present paper by Tinggard et al. provides a comprehensive approach of physical activity assessment in patients with chronic heart failure compared to age- and sex-matched controls. Importantly, the authors correlated measures of physical activity, using a double-validated 3D accelerometer being worn for 7 days, with objective measures of muscle mass and strength, functional capacity during spiroergometry and 6-min walk test, in addition to NYHA class, NT-proBNP levels and LV ejection fraction. The authors used raw data to calculate average acceleration and intensity gradients, as recommended by [5, 6], and assessed daily average acceleration, time spent in moderate to vigorous and light physical activity as well as time spent in sedentary activity.

Of note, the applied cutoffs for the different activity levels were previously established in older or chronically ill adults, but not in HF patients [7]. The authors show that the daily average acceleration is significantly reduced in patients with HFrEF compared to controls, with a mean acceleration that was lower than the acceleration found in a recent data analysis from the UK biobank [8]. However, this may be related to the present restriction to HFrEF patients. Additional analyses in the present manuscript show that patients with chronic HFrEF have a pronounced reduction of the time spent in moderate to vigorous physical activity, which is compensated by an increase in sedentary time, in comparison to healthy controls.

Interestingly, there was only a rather moderate correlation between physical activity and established functional tests, represented by peak VO2 and 6-min walk test. By multivariate analysis, 6MWT and peakVO2 predicted the average acceleration and time in moderate to vigorous activity. Therefore, it is tempting to speculate that these parameters can also predict long-term prognosis in HF patients, which would be easier compared to classical functional testing because data acquisition could be performed automatically during extended time periods in the background. Interestingly, after division of patients into two groups with either low or high physical activity, patients with low physical activity demonstrated a higher prevalence of low muscle mass. However, the absolute muscle strength, as assessed by handgrip exercise or knee-extensor strength, did not show any significant differences between HF patients with either a low or a high physical activity profile. It remains therefore speculative whether a low physical activity is a characteristic of the individual patient leading to a high likelihood of cardiovascular disease development or a phenotypic characteristic of the disease, in this case heart failure. In the present paper, patients with low physical activity more frequently suffered from diabetes type 2, which can be effectively prevented by physical activity [9].

Recent studies have highlighted the critical role of daily step count, intensity and step speed in reducing the risk of cardiovascular diseases (CVD) and mortality [10–12]. These studies consistently show that higher daily step counts, especially when combined with higher step frequency, are associated with lower risks of all-cause and cardiovascular mortality. Moreover, increasing the intensity of these steps, measured by walking speed or cadence, enhances cardiovascular benefits, underscoring the importance of not just the quantity, but also the quality of physical activity [10, 11].

The emphasis on everyday activities such as walking highlights a practical, accessible strategy for CVD prevention and management. This is particularly relevant for individuals with chronic heart failure, where high-intensity exercise may not be feasible. Encouraging patients to increase their daily steps, even in small increments, can yield substantial health benefits, including improved lipid profiles, blood pressure regulation and enhanced insulin sensitivity. In addition to the direct cardiovascular benefits, regular walking and increased physical activity levels are associated with better mental health, improved sleep quality and reduced stress levels. These factors collectively contribute to the overall well-being, further reinforcing the importance of maintaining an active lifestyle. The use of wearable devices and smartphone apps to monitor step count and intensity provides a valuable tool for both individuals and healthcare providers to track and encourage physical activity, making it easier to set and achieve health goals.

In future studies, it would be interesting to see the pulmonary hemodynamic response to different levels of daily physical activity in HF patients, because impaired ventricular compliance is associated with increased pulmonary artery pressures, leading to dyspnea and limited exercise capacity. Finally, whereas functional tests have established prognostic thresholds that trigger further treatment like evaluation for cardiac transplantation, daily physical activity levels did not yet demonstrate comparable prognostic accuracy. This may be in part be due to different applied accelerometers, worn at different places in different patient cohorts for different time periods. Therefore, future studies need to establish the prognostic cutoffs from raw data for average acceleration, intensity gradients and the heart failure-specific cutoffs for sedentary, moderate or vigorous activity. This would also have the advantage that the measurements from different accelerometer data would be comparable across studies and can also be used as read-out for clinical trials [13]. Finally, the anticipated use of accelerometry in heart failure patients is not restricted to research purposes. Because more medical devices like pacemakers or ICDs, but also smart watches and mobile phones contain validated 3D accelerometry, these data could and should be used for patient-tailored rehabilitation, training and motivation for physical activity when user-friendly apps are available.
